# Maternal Pregnancy Levels of Polychlorinated Biphenyls and Risk of Hypospadias and Cryptorchidism in Male Offspring

**DOI:** 10.1289/ehp.0800389

**Published:** 2009-04-20

**Authors:** Katherine A. McGlynn, Xuguang Guo, Barry I. Graubard, John W. Brock, Mark A. Klebanoff, Matthew P. Longnecker

**Affiliations:** 1 Division of Cancer Epidemiology and Genetics, National Cancer Institute, National Institutes of Health, Department of Health and Human Services, Bethesda, Maryland, USA; 2 Westat, Inc., Durham, North Carolina, USA; 3 National Center for Environmental Health, Centers for Disease Control and Prevention, Atlanta, Georgia, USA; 4 Division of Epidemiology, Statistics, and Prevention Research, Eunice Kennedy Shriver National Institute of Child Health and Human Development, National Institutes of Health, Department of Health and Human Services, Rockville, Maryland, USA; 5 Epidemiology Branch, National Institute of Environmental Health Sciences, National Institutes of Health, Department of Health and Human Services, Research Triangle Park, North Carolina, USA

**Keywords:** cryptorchidism, hypospadias, polychlorinated biphenyls, testicular dysgenesis syndrome

## Abstract

**Background:**

The etiologies of the male urogenital anomalies cryptorchidism and hypospadias are poorly understood. It has been suggested, however, that *in utero* hormone levels may be related to risk. Endocrine-disrupting chemicals, including polychlorinated biphenyl (PCB) compounds, may alter hormone levels and thereby affect the fetus.

**Objectives:**

To examine whether *in utero* PCB exposure is related to cryptorchidism and hypospadias, we examined PCB levels among pregnant women enrolled in the Collaborative Perinatal Project (CPP).

**Methods:**

The CPP enrolled pregnant women at 12 U.S. medical centers between 1959 and 1965. For the present research, we analyzed third-trimester serum samples from the mothers of 230 sons with cryptorchidism, 201 sons with hypospadias, and 593 sons with neither condition. We estimated adjusted odds ratios (ORs) and 95% confidence intervals (CIs) using logistic regression and examined the associations of each anomaly with individual PCB congener levels, sum of PCBs, and several functional groupings of PCBs.

**Results:**

In general, the ORs for cryptorchidism or hypospadias showed no notable associations with individual PCB congener levels or functional groupings of PCBs. However, the ORs and 95% CIs for the sum of PCBs associated with hypospadias were as follows: 0–1.9 μg/L, reference group; 2–2.9 μg/L, OR = 1.57, 95% CI, 1.05–2.34; 3–3.9 μg/L, OR = 1.45, 95% CI, 0.90–2.34; and ≥ 4.0 μg/L, OR = 1.69, 95% CI, 1.06–2.68; *p*-value for trend = 0.08.

**Conclusions:**

Given the large number of associations examined, these findings do not strongly support the hypothesis that PCBs are associated with cryptorchidism or hypospadias. Because population serum PCB levels at the time of sample collection were considerably higher than levels at present, it is unlikely that current PCB exposure is related to the development of either anomaly.

The hormone hypothesis of male reproductive disorders suggests that both endogenous and exogenous hormones might be risk factors for cryptorchidism, hypospadias, impaired spermatogenesis, and testicular cancer (Sharpe 2003). Exogenous hormonal exposures involve a variety of endocrine-disrupting chemicals whose effects range from estrogenic to anti-estrogenic, androgenic, and antiandrogenic. Evidence in support of the hypothesis has consisted largely of animal data (Toppari et al. 1996) and temporal trends (Paulozzi 1999) in the prevalence of the so-called testicular dysgenesis syndrome (TDS) conditions. Human studies of the relationship between endocrine-disrupting chemicals and the TDS disorders have been relatively few.

Polychlorinated biphenyl (PCB) compounds are ubiquitous, stable, environmental pollutants that were widely used in developed countries between 1929 and 1977. Even after they were banned, PCBs continued to be used in “closed” source applications such as electrical capacitors and transformers. Levels of PCBs in humans have declined since the 1960s but remain detectable in most segments of the population (Ross 2004). To date, only four published studies have examined the association between PCBs and either cryptorchidism or hypospadias. Two case–control studies of PCBs and cryptorchidism reported no relationship with risk (Hosie et al. 2000; Mol et al. 2002), whereas a third offered measured support (Brucker-Davis et al. 2008). One ecologic study of PCBs and hypospadias reported an inverse association (Giwercman et al. 2006). Because the prior studies may have been too small to detect statistically significant differences, the present study was conducted among a large, well-described population in which the serum samples were collected at a time when PCB levels in the United States were higher.

## Materials and Methods

### Study population

The Collaborative Perinatal Project (CPP) was a prospective study of neurologic disorders and other conditions in children (Niswander and Gordon 1972). Pregnant women were enrolled between 1959 and 1965 at 12 U.S. medical centers located in Baltimore, Maryland; Boston, Massachusetts; Buffalo, New York; Memphis, Tennessee; Minneapolis, Minnesota; New Orleans, Louisiana; New York City (two centers); Philadelphia, Pennsylvania; Portland, Oregon; Providence, Rhode Island; and Richmond, Virginia. Eleven centers recruited participants from the prenatal clinics of a university hospital, whereas one (Buffalo) recruited from 13 private obstetric practices. Participant selection varied among the medical centers. Women were ineligible if they were incarcerated, planned to leave the area on delivery, or planned to place their child for adoption or if they delivered on the day they were recruited for the study. The characteristics of the women in the sample were, at registration, essentially the same as those in the sampling frame (Niswander and Gordon 1972). Four percent of the participants were lost to follow-up before delivery. Once enrolled, the women’s nonfasting blood was drawn approximately every 8 weeks during pregnancy, at delivery, and at 6 weeks postpartum. Sera were stored in glass vials at −20°C with no recorded thaws. Approximately 42,000 women were enrolled, and 55,000 children were born in the study. The children were systematically assessed for the presence of birth defects and other outcomes at birth and through 7 years of age. Follow-up to 7 years of age was completed for about 75% of children born into the study.

For the present study, we employed a nested case–control design to examine the association between maternal serum PCB levels and risk of cryptorchidism and hypospadias among sons. To be eligible for inclusion in the present study, the mother must have given birth to a singleton, live-born male infant and must have had a 3-mL aliquot of third-trimester maternal serum available. We defined cryptorchidism as having had a diagnosis of undescended testis(es) at any time during the first year of life. The diagnosis was made by pediatricians based on serial examinations that included inspection and palpation of the genitalia. Boys first noted in the medical record to have undescended testis(es) after the first year of life were not considered cryptorchid because they may have had retractile testes. Hypospadias was defined as having a diagnosis any time during the first 7 years of life. Degree of hypospadias was not noted in the medical records.

Among the 28,444 boys in the CPP, 267 were not live born and 441 were not singletons. No blood sample was available from the mothers of 5,389 boys. Among the eligible 22,347 boys, there were 241 cases of cryptorchidism and 214 cases of hypospadias. The five boys with both cryptorchidism and hypospadias were included in each group for the analysis. From the pool of eligible mothers, 599 were selected at random so that the control:case ratio would be > 2:1 for each condition.

Among the boys diagnosed as cryptorchid during the first year of life, the study records indicated that the testicles were descended at birth in 103, suggesting that these boys may have had acquired, rather than congenital, undescended testis (Barthold and Gonzalez 2003). To evaluate whether a relationship with PCBs varied by type of cryptorchidism, we considered the boys with testis descended at birth separately in a sensitivity analysis. Among the boys with undescended testicle(s) at birth (*n* = 138), all but one also had a subsequent observation of cryptorchidism in at least one of the three subsequent examination (ages 4 months, 1 year, 7 years) or documentation of orchidopexy.

The socioeconomic index calculated for participants in the CPP was the mean of the three percentile scores: education of head of the household, occupation of head of the household or chief wage earner, and family income. The score used to calculate the percentile for an occupation was based on the percentiles of education and income among persons with the same occupation (Myrianthopoulos and French 1968). Verbal consent to participate was elicited from all mothers (Hardy 2003).

### Laboratory assays

Serum levels of 11 PCBs were measured at the Centers for Disease Control and Prevention after solid-phrase extraction cleanup and dual-column gas chromatography using electron capture detection (Brock et al. 1996). The congeners, designated by International Union of Pure and Applied Chemistry code, were PCB-28, PCB-52, PCB-74, PCB-105, PCB-118, PCB-138, PCB-153, PCB-170, PCB-180, PCB-194, and PCB-203. The proportion of PCBs in each sample recovered by extraction was approximately 60%. The results shown are unadjusted for recovery. The between-assay coefficient of variation was 19% at 3.49 μg PCB. A laboratory result was not obtained for 3% of samples because the measured value did not meet the quality-control standards for acceptance. Thus, the PCB results were not available for 30 samples. Because PCBs are lipid soluble and because serum contains a variable amount of lipid, we measured serum cholesterol and triglycerides using standing enzymatic assays.

In addition to analyzing each congener separately, we examined four groupings of congeners. The Wolff groupings (Wolff et al. 1997) were as follows: Wolff group 1A, potentially estrogenic, weak phenobarbital inducers, not persistent congeners (PCB-52); Wolff group 2A, potentially antiestrogenic and immunotoxic, dioxin-like, non-*ortho*– and mono-*ortho*–substituted, moderately persistent congeners (PCB-74, PCB-105, PCB-118); Wolff group 2B, limited dioxin activity, di-*ortho* substituted, persistent congeners (PCB-138, PCB-170) and Wolff group 3, phenobarbital inducers, CYP1A and CYP2B inducers, and persistent congeners (PCB-153, PCB-180, PCB-203). The toxic equivalency factor (TEF) groupings (Ahlborg et al. 1994) were TEF mono-*ortho* (PCB-105, PCB-118), and TEF di-*ortho* (PCB-170, PCB-180). The uridine diphosphate-glucurono-syl transferase (UDP-GT) inducer, cytochrome P450 1A (CYP1A), and CYP2B inducer grouping (Chevrier et al. 2007) was PCB-118 and PCB-180. The molecular-weight groups were low (PCB-28, PCB-52, PCB-74, PCB-105, PCB-118) and high (PCB-138, PCB-153, PCB-170, PCB-180, PCB-194, PCB-203).

### Statistical methods

We converted PCBs to molar concentrations (PCB in micrograms per liter divided by the congener’s molecular weight) and then analyzed on a continuous scale. Five congeners had missing values (PCB-28, *n* = 2; PCB-74, *n* = 26; PCB-118, *n* = 42; PCB-138, *n* = 22; PCB-180, *n* = 2), mainly because the measured value did not meet the quality control standards for acceptance (Ballschmiter et al. 1992). We estimated the change in log odds of having cryptorchidism or hypospadias per millimole increase in PCB level using conditional logistic regression, conditioned on study center (12 strata). Models were adjusted for serum triglycerides and cholesterol as continuous variables and serum *p*,*p’*-dichloro-diphenyldichloroethylene (*p*,*p’*-DDE) as a five-stratum categorical variable.

We evaluated confounding by comparing the hazard ratio of the baseline model (including sum of PCBs, triglycerides, cholesterol, and *p*,*p’*-DDE) to the hazard ratio of the model that also included the possible confounder. We modeled sum of PCBs both as a continuous variable and as a categorical variable and used results of both analyses to determine whether the factor was a confounder. If the hazard ratio per microgram per liter of PCB or the hazard ratio for the contrast of highest-to-lowest PCB strata changed by 15% or more, the factor was considered a confounder. The factors considered as possible confounders were race, season of birth, maternal age, maternal parity, socioeconomic index, prepregnancy body mass index, weight gain during pregnancy, smoking during pregnancy, hyperemesis gravidarum, gestational hypertension, age at menarche, history of infertility, menstrual cycle irregularity, estrogen use during pregnancy, and progester-one use during pregnancy. Similarly, we considered the effects of adjustment for preterm birth, birth weight, placental weight, and small-for-gestational-age even though these were potentially intermediate variables. None of the factors considered, however, changed the hazard ratios by ≥ 15%. Using a similar approach, but with cross-product terms, we evaluated effect modification by maternal age, race, smoking, prepregnancy body mass index, triglycerides, cholesterol, serum *p*,*p’*-DDE, gestational hypertension, socioeconomic index, and study center. We supplemented evaluation of effect modification by categorical variables with more than two categories by comparing the model fit statistics for models with and without the cross-product terms. If the *p*-value associated with the interaction term based on the likelihood ratio test had a value ≤ 0.10, the degree of potential effect modification was further considered by examining tables stratified by the potentially modifying factor(s). We further evaluated sum of PCBs by forming ordinal categories and including corresponding dummy variables in the logistic regression analysis to estimate adjusted odds ratios (ORs). We performed trend tests across categories by including a single independent variable with integer scores corresponding to the categories testing the significance of the regression coefficient. All statistical analyses were conducted using the SAS statistical software package, version 9.1 (SAS Institute Inc., Cary, NC). All *p*-values were two-sided.

## Results

Based on the distribution of the controls, the study population was 46% white, 48% black, and 5.6% other racial/ethnic groups (primarily Hispanic and Asian) (Table 1). Birth weight and gestational age medians were typical of healthy births. The mothers were a median age of 22 years and had a median socioeconomic index (mean of three percentile scores: education of head of household, occupation of head of household or chief wage earner, and family income) of 4.5, which was just under of the median of 5.0 for the United States population in the 1960s. About 31% of the mothers were primiparas. Compared with the control boys, the boys with cryptorchidism and hypospadias were more likely to be white, to be born prematurely, and to be born small for gestational age (birth weight below 10th percentile). The boys with hypospadias also had a lower median birth weight than did the control boys. A previous report has described the study population in detail (Longnecker et al. 2002).

Table 2 shows median maternal PCB levels, lipid levels, and DDE levels for each of the study groups. The median level of sum of PCBs was slightly lower in the controls (2.7 μg/L), whereas the median levels of *p*,*p’*-DDE (24.5 μg/L) and total cholesterol (234 μg/L) were slightly higher. The median level of triglycerides in the controls (204 μg/L) was intermediate to the two case groups.

Table 3 shows the adjusted ORs for the PCB congener-specific analyses, conditioned on study center and adjusted for triglyc-erides, cholesterol, and *p*,*p’*-DDE level. For cryptorchidism, the ORs ranged from 1.03 (PCB-118) to 1.79 (PCB-203), and none were statistically significant. For hypospadias, the ORs ranged from 0.80 (PCB-52) to 1.99 (PCB-203), and as with cryptorchidism, none were statistically significant.

Table 3 also shows the adjusted ORs for groupings of PCBs. We found no significant increase in risk of either cryptorchidism or hypospadias with increasing level of sum of PCBs [cryptorchidism: OR = 1.01, 95% confidence interval (CI), 0.99–1.04; hypospadias: OR = 1.01, 95% CI, 0.98–1.04]. Similarly, we found no significant increases in risk of either outcome with any of the Wolff groupings (Wolff et al. 1997), TEF groupings (Ahlborg et al. 1994), or enzyme inducer (Chevrier et al. 2007) groupings. Because the ORs in the congener-specific analysis tended to be higher among the higher-molecular-weight congeners, we also analyzed PCBs stratified by molecular weight. The analyses did not detect statistically significant associations with either cryptorchidism (low: OR = 1.02, 95% CI, 0.97–1.06; high: OR = 1.04, 95% CI, 0.98–1.09) or hypospadias (low: OR = 1.00, 95% CI, 0.95–1.06; high: OR = 1.04, 95% CI, 0.98–1.10).

Because the examinations of cryptorchidism and hypospadias with the sum of PCBs approached statistical significance, we also analyzed risk by PCB category (Table 4). Cryptorchidism was not significantly related to PCB level in any category, and the test for trend was not statistically significant (*p* for trend = 0.19). The results of a similar analysis that modeled sum of PCBs on a lipid basis in quintiles produced the same conclusions (data not shown). Although the trend in risk of hypospadias approached significance (*p* for trend = 0.08) and the ORs for all categories were greater than unity, we found, overall, no linear relationship. The examination of effect modification showed that no factor significantly altered the results of the baseline models (data not shown). A final examination of cryptorchidism excluded the boys (*n* = 103) whose testicles were initially descended at birth. The results of that analysis (data not shown), however, did not differ from the analysis that included all boys with cryptorchidism.

## Discussion

The results of the present study do not support the hypothesis that *in utero* PCB exposure increases the risk of hypospadias or cryptorchidism. Because the present study samples were collected in the 1960s, when PCB exposure was substantially greater than at present, the results also suggest that current low-level PCB exposure is unlikely to be related to the development of either condition.

With the inclusion of > 1,000 participants, the present study is by far the largest to have examined the relationship between PCBs and cryptorchidism or hypospadias. Although the prior literature is not extensive, three studies of cryptorchidism and PCBs and one study of hypospadias and PCBs have been published. Mol et al. (2002) examined cord blood PCB levels in a population known to have high PCB exposure via fish consumption. Studying 196 Faroe Island boys born between 1986 and 1987, the investigators found no relationship between PCB levels and cryptorchidism. A similar conclusion was reached by Hosie et al. (2000). Studying adipose PCB levels in 18 German boys with cryptorchidism and 30 control boys, the investigators found no difference in PCB levels. A third study examined PCB levels in both cord blood and breast milk in a French population (Brucker-Davis et al. 2008). The comparison of 78 boys diagnosed at birth with cryptorchidism and 86 control boys found a significant association with breast milk PCB levels but not with cord blood PCB levels. When they restricted the analysis to the boys who remained cryptorchid at 3 months, however, the relationship with breast milk PCB levels was no longer significant. The only prior study of hypospadias and PCB levels reported to date was an ecologic study in Greenland. The investigators found a low prevalence rate of hypospadias despite high levels of PCBs in the population (Giwercman et al. 2006), suggesting that PCBs might be inversely, rather than directly, associated with risk of hypospadias.

PCBs have been suspected of being related to male urogenital anomalies principally because some congeners and metabolites display weak estrogenic activity. There is no evidence in the present results, however, that estrogenic activity is a risk factor. The lower-molecular-weight congeners have greater estrogenic activity than the higher-molecular-weight congeners (Wolff et al. 1997), yet it was the higher-molecular-weight congeners that had the greatest, although nonsignificant, ORs. In addition, it has been noted that the estrogenic potency of PCBs is extremely low compared with naturally occurring estrogens, so exposure may have little ability to affect the *in utero* estrogenic milieu (Safe 1995). In addition to estrogenic effect, there is evidence that PCBs also may have anti androgenic effects, although the data are few and inconsistent (Ulbrich and Stahlmann 2004). Reduced anogenital distance has been reported, as has interference by PCB-138 with androgen receptor–mediated effects (Bonefeld-Jorgensen et al. 2001; Portigal et al. 2002; Schrader and Cooke 2003). Coplanar PCBs may also have antiandrogenic effects (Mocarelli et al. 2008). Although no coplanar PCBs were measured in the present study, their levels generally correlate well with sum of PCB levels (Gladen et al. 1999; Longnecker et al. 2000).

If PCBs are able to alter the maternal hormonal milieu, it is possible that they would also be related to the TDS disorders (Skakkebaek et al. 2001) that become evident in adulthood: impaired spermatogenesis and testicular cancer. More studies have examined the relationship of PCBs with the former than the latter. In general, the results of the PCB–fertility studies are somewhat equivocal. Several studies have found statistically significant associations with impaired sperm parameters (Hauser et al. 2003; Richthoff et al. 2003; Rozati et al. 2002), whereas others have found no association (Weiss et al. 2006) or associations only in subsets of their populations (Bush et al. 1986; Dallinga et al. 2002; Rignell-Hydbom et al. 2004). In contrast, several studies have reported direct associations between PCB levels and fertility (Cok et al. 2008; Ensslen et al. 1990). The summary of the international INUENDO study of PCBs and fertility in four populations, however, concluded that PCBs did not appear to affect fertility or to have direct hormone-like activity (Bonde et al. 2008). To date, the sole published study of testicular cancer and PCBs found no association with sum of PCBs, estrogenic PCBs, or enzyme-inducing PCBs (Hardell et al. 2003). Taken as a group, then, at the present time, there is little evidence that the TDS disorders are related to PCB exposure.

Several potential weaknesses of the present study merit consideration. A first consideration is that the assays were run on stored, rather than fresh, serum samples. PCB levels, however, are quite stable over time, as has been demonstrated in samples stored for 15 years (Lunden and Noren 1998; Noren 1988). In addition, the cholesterol and triglyceride levels, as reported in Table 2, were in the expected range, suggesting that substantial degradation had not occurred. A second potential weakness is that determination of one of the outcomes, cryptorchidism, can be problematic. A single determination of testicular descent in the delivery room is not always accurate. Testes descend late in the third trimester and are not uncommonly undescended at birth. In addition, it is now generally acknowledged that “acquired undescended testes” does occur, although the age at occurrence remains a matter of some debate (Barthold 2008). However, the CPP examined the children systematically over time to ascertain congenital anomalies (Myrianthopoulos and Chung 1974). As a result, the rates of both cryptorchidism and hypospadias reported in the CPP were higher (Myrianthopoulos and Chung 1974) than they were other U.S. studies (Paulozzi 1999). For the present study, the ratio of hypospadias to cryptorchidism was somewhat higher than might be expected because we included hypospadias diagnosed up until 7 years of age, whereas we excluded cryptorchid cases diagnosed after 1 year. A final limitation of the study was that the medical records did not note degree of hypospadias among the boys affected.

In conclusion, the results of the present study do not support an association between *in utero* PCB exposure and either cryptorchid-ism or hypospadias. PCBs are just one type of environmental endocrine modulator, however. Whether other endocrine modulators are related to risk is unclear and should be examined in other studies.

## Figures and Tables

**Table 1:** Characteristics of mothers and sons according to the son’s case–control status, CPP, 1959–1965.

Characteristic	Cryptorchidism (*n* = 230)	Hypospadias (*n* = 201)	Control (*n* = 593)
Race (%)			
White	57.0	49.3	46.0
Black	41.3	44.8	48.4
Other	1.7	6.0	5.6
Gestation (week)			
Median (Q1, Q3)	39 (38, 41)	39 (38, 41)	39 (38, 40)
Preterm birth (%)	16.6	19.5	14.0
Birth weight (g)			
Median (Q1, Q3)	3,260 (2,835, 3,629)	3,147 (2,665, 3,487)	3,260 (2,948, 3,600)
Small for gestational age (%)	9.1	17.8	4.9
Maternal age (years)			
Median (Q1, Q3)	24 (21, 30)	24 (20, 29)	22 (20, 28)
Previous live births (%)			
0	27.0	30.8	30.9
1	22.2	21.4	22.5
≥ 2	50.9	47.8	46.6
Socioeconomic index			
Median (Q1, Q3)	4.7 (3.3, 6.3)	4.3 (3.0, 6.2)	4.5 (3.3, 6.0)
Prepregnancy body mass index			
Median (Q1, Q3)	22.2 (20.3, 25.0)	21.8 (19.3, 24.1)	22.2 (20.0, 24.9)
Gestational hypertension (%)	5.9	6.5	6.4
Study Center (%)			
Boston, MA	31.3	25.4	23.6
Buffalo, NY	7.0	3.5	3.5
New Orleans, LA	6.5	3.5	4.7
New York City, NY*a*	3.0	4.5	3.2
Baltimore, MD	6.1	6.5	7.6
Richmond, VA	7.0	5.5	5.7
Minneapolis, MN	3.5	4.0	5.2
New York City, NY*b*	1.7	6.0	7.8
Portland, OR	4.3	5.5	7.1
Philadelphia, PA	14.8	23.9	18.5
Providence, RI	11.3	10.0	5.9
Memphis, TN	3.5	2.0	7.1

a Columbia-Presbyterian Medical Center.

b New York Medical College

**Table 2:** Maternal serum values by son’s case–control status, CCP, 1959–1965.

	Cryptorchidism (*n* = 230)	Hypospadias (*n* = 201)	Control (*n* =593)
Total cholesterol (ug/L)			
Median (Q1, Q3)	231 (190, 272)	229 (190, 275)	234 (195, 279)

Triglycerides (ug/L)			
Median (Q1, Q3)	207.5 (163, 258)	187 (152, 253)	204 (159, 259)

DDE (ug/L)			
Median (Q1, Q3)	23.6 (15.9, 35.3)	23.9 (16.2, 34.4)	24.5 (16.7, 37.2)

Sum of PCBs with imputed congener (ug/L)			
Median (Q1, Q3)	2.8 (2.0, 3.9)	2.9 (2.1, 4.2)	2.7 (1.8, 3.8)

**Table 3:** Adjusted ORs (95% CIs) for cryptorchidism and hypospadias in relation to maternal PCB levels (mmol/L), CPP, 1959–1965.

	Percentiles among controls				Cryptorchidism	Hypospadias
	25th	50th	75th	95th	Adjusted*a* OR (95% CI)	Adjusted*a* OR (95% CI)
PCB congener						
PCB-28	0.39	0.70	0.97	1.79	1.07 (0.86–1.34)	1.07 (0.86–1.32)
PCB-52	0.00	0.00	0.00	0.51	1.11 (0.81–1.52)	0.80 (0.42–1.52)
PCB-74	0.51	0.79	1.13	2.16	1.04 (0.87–1.25)	1.03 (0.84–1.26)
PCB-105	0.00	0.34	0.52	1.19	1.11 (0.82–1.50)	1.03 (0.69–1.53)
PCB-118	1.13	1.65	2.39	5.21	1.03 (0.95–1.12)	0.99 (0.88–1.11)
PCB-138	1.16	1.61	2.36	3.85	1.09 (0.94–1.25)	1.08 (0.93–1.26)
PCB-153	1.22	1.69	2.52	4.02	1.11 (0.97–1.27)	1.12 (0.98–1.30)
PCB-170	0.00	0.23	0.38	0.66	1.07 (0.59–1.94)	1.34 (0.73–2.46)
PCB-180	0.35	0.56	0.83	1.39	1.19 (0.84–1.69)	1.28 (0.89–1.84)
PCB-194	0.00	0.00	0.21	0.37	1.42 (0.54–3.72)	1.41 (0.48–4.19)
PCB-203	0.00	0.00	0.21	0.42	1.79 (0.71–4.52)	1.99 (0.75–5.28)
Sum of PCBs	5.29	7.86	10.92	19.48	1.01 (0.99–1.04)	1.01 (0.98–1.04)
UPD-GT inducers	1.44	2.15	3.12	6.19	1.03 (0.96–1.11)	1.01 (0.92–1.11)
Wolff groups						
1A	0.00	0.00	0.00	0.51	1.11 (0.81–1.52)	0.80 (0.42–1.52)
2A	1.78	2.61	3.75	8.06	1.02 (0.97–1.07)	1.00 (0.94–1.07)
2B	1.22	1.75	2.66	4.41	1.07 (0.94–1.21)	1.07 (0.94–1.22)
3	1.59	2.30	3.49	5.59	1.08 (0.98–1.18)	1.09 (0.98–1.20)
TEF Groups						
Mono-*ortho*	1.19	1.93	2.85	6.13	1.02 (0.96–1.10)	0.99 (0.91–1.09)
Di-*ortho*	0.38	0.73	1.21	1.97	1.09 (0.87–1.38)	1.16 (0.92–1.48)
Molecular weight groups						
Low	2.31	3.36	4.68	9.98	1.02 (0.97–1.06)	1.00 (0.95–1.06)
High	2.8	4.21	6.32	10.27	1.04 (0.98–1.09)	1.04 (0.98–1.10)

a Results are from conditional logistic regression, conditioned on study center and adjusted for serum DDE level (5 strata), triglycerides, and cholesterol. OR is change in lnOR per mmol/L increase in PCB.

**Table 4:** Adjusted ORs (95% CIs) for cryptorchidism and hypospadias in relation to total PCB level in maternal serum, CPP, 1959–1965.

Total PCBs (ug/L)	Control (no.)	Cryptorchism		Hypospadias	
		No.	Adjusted*a* OR (95% CI)	No.	Adjusted*a* OR (95% CI)
0–1.9	180	55	1.00	41	1.00
2–2.9	171	72	1.27 (0.88–1.83)	67	1.57 (1.05–2.34)
3–3.9	111	48	1.32 (0.86–2.02)	38	1.45 (0.90–2.34)
≥ 4.0	131	55	1.41 (0.90–2.20)	55	1.69 (1.06–2.68)
*p*-Trend*b*			0.19		0.08

a Adjusted for serum DDE concentration (five categories), triglycerides, and cholesterol.

b Ordinal test across four categories, using the median value within each group.
